# Entropy of mitochondrial DNA circulating in blood is associated with hepatocellular carcinoma

**DOI:** 10.1186/s12920-019-0506-7

**Published:** 2019-06-06

**Authors:** David S. Campo, Vishal Nayak, Ganesh Srinivasamoorthy, Yury Khudyakov

**Affiliations:** 1 Division of Viral Hepatitis, Centers for Disease Control and Prevention, Atlanta, GA USA; 2 Office of Advanced Molecular Detection, Centers for Disease Control and Prevention, Atlanta, GA USA; 3 CSRA, Inc, Corporate Blvd NE, Atlanta, GA USA

**Keywords:** Mitochondria, Liquid biopsy, Machine learning, Entropy

## Abstract

**Background:**

Ultra-Deep Sequencing (UDS) enabled identification of specific changes in human genome occurring in malignant tumors, with current approaches calling for the detection of specific mutations associated with certain cancers. However, such associations are frequently idiosyncratic and cannot be generalized for diagnostics. Mitochondrial DNA (mtDNA) has been shown to be functionally associated with several cancer types. Here, we study the association of intra-host mtDNA diversity with Hepatocellular Carcinoma (HCC).

**Results:**

UDS mtDNA exome data from blood of patients with HCC (*n* = 293) and non-cancer controls (NC, *n* = 391) were used to: (i) measure the genetic heterogeneity of nucleotide sites from the entire population of intra-host mtDNA variants rather than to detect specific mutations, and (ii) apply machine learning algorithms to develop a classifier for HCC detection. Average total entropy of HCC mtDNA is 1.24-times lower than of NC mtDNA (*p* = 2.84E-47). Among all polymorphic sites, 2.09% had a significantly different mean entropy between HCC and NC, with 0.32% of the HCC mtDNA sites having greater (*p* < 0.05) and 1.77% of the sites having lower mean entropy (*p* < 0.05) as compared to NC. The entropy profile of each sample was used to further explore the association between mtDNA heterogeneity and HCC by means of a Random Forest (RF) classifier The RF-classifier separated 232 HCC and 232 NC patients with accuracy of up to 99.78% and average accuracy of 92.23% in the 10-fold cross-validation. The classifier accurately separated 93.08% of HCC (*n* = 61) and NC (*n* = 159) patients in a validation dataset that was not used for the RF parameter optimization.

**Conclusions:**

Polymorphic sites contributing most to the mtDNA association with HCC are scattered along the mitochondrial genome, affecting all mitochondrial genes. The findings suggest that application of heterogeneity profiles of intra-host mtDNA variants from blood may help overcome barriers associated with the complex association of specific mutations with cancer, enabling the development of accurate, rapid, inexpensive and minimally invasive diagnostic detection of cancer.

## Background

Cancer is the leading cause of morbidity and mortality worldwide, with the estimated 14 million new cases and 8.2 million cancer-related deaths in 2012, and this number is predicted to rise by ~ 70% over the next two decades [1]. Successful clinical management of cancer patients is largely contingent on early tumor detection and accurate assessment of treatment efficacy [2]. Currently, the standard diagnostic procedure for cancer is histological analysis of tissue biopsy. However, biopsies have several disadvantages, as they are invasive, costly and time-consuming. Only highly trained pathologists can perform histological detection and characterization of cancer from the sampled tissue. In addition, although generally safe, biopsies may cause complications such as bleeding, infection and accidental injury to adjacent structures [2].

Further improvement of cancer patient care greatly depends on development of accurate, minimally invasive, inexpensive and rapid diagnostic techniques. Recent progress in the identification of cancer biomarkers opened a new field of cancer diagnostics (see [2] for a review). The rapid, cheap and non-invasive nature of the “liquid biopsy” has the potential to bring fundamental change to cancer care by allowing for a repeat sampling and testing of blood for the early disease detection and effective monitoring of treatment responses [3].

Tumors shed nucleic acids into blood, a phenomenon that was exploited since the early discovery of cancer-related DNA mutations [4–7]. Screening of the whole human genome, the exome or mitochondrial DNA allows for the detection of mutant DNA species associated with different malignant tumors (see [3] for a review). Detection of tumor DNA circulating in blood provides a direct measure of cancer rather than an indirect assessment of the effects of cancer. However, low concentration of the tumor DNA in blood hampers its use in diagnostics. Recently, ultra-deep sequencing (UDS) has been applied to the efficient detection of the tumor DNA [8], thus significantly facilitating early cancer detection in asymptomatic individuals. Such mutant DNA species can be detected even at a very low concentration in blood of patients [9]. However, the complex and variable genetic nature of cancer in each patient often hinders the identification of mutations suitable for cancer diagnostics (see [3] for a review).

Here, we show that heterogeneity profiles of the intra-host mtDNA population are strongly associated with Hepatocellular Carcinoma (HCC). The small size of mtDNA is especially suitable for the accurate assessment of such profiles, application of which to the HCC detection overcomes the often-idiosyncratic association of specific mutations to cancer. The findings in this study suggest that genetic diversity of intra-host mtDNA in blood may serve as a generalizable marker for the accurate, rapid, inexpensive and minimally invasive diagnostic detection of cancer.

## Methods

### Datasets

**Fig. 1:**
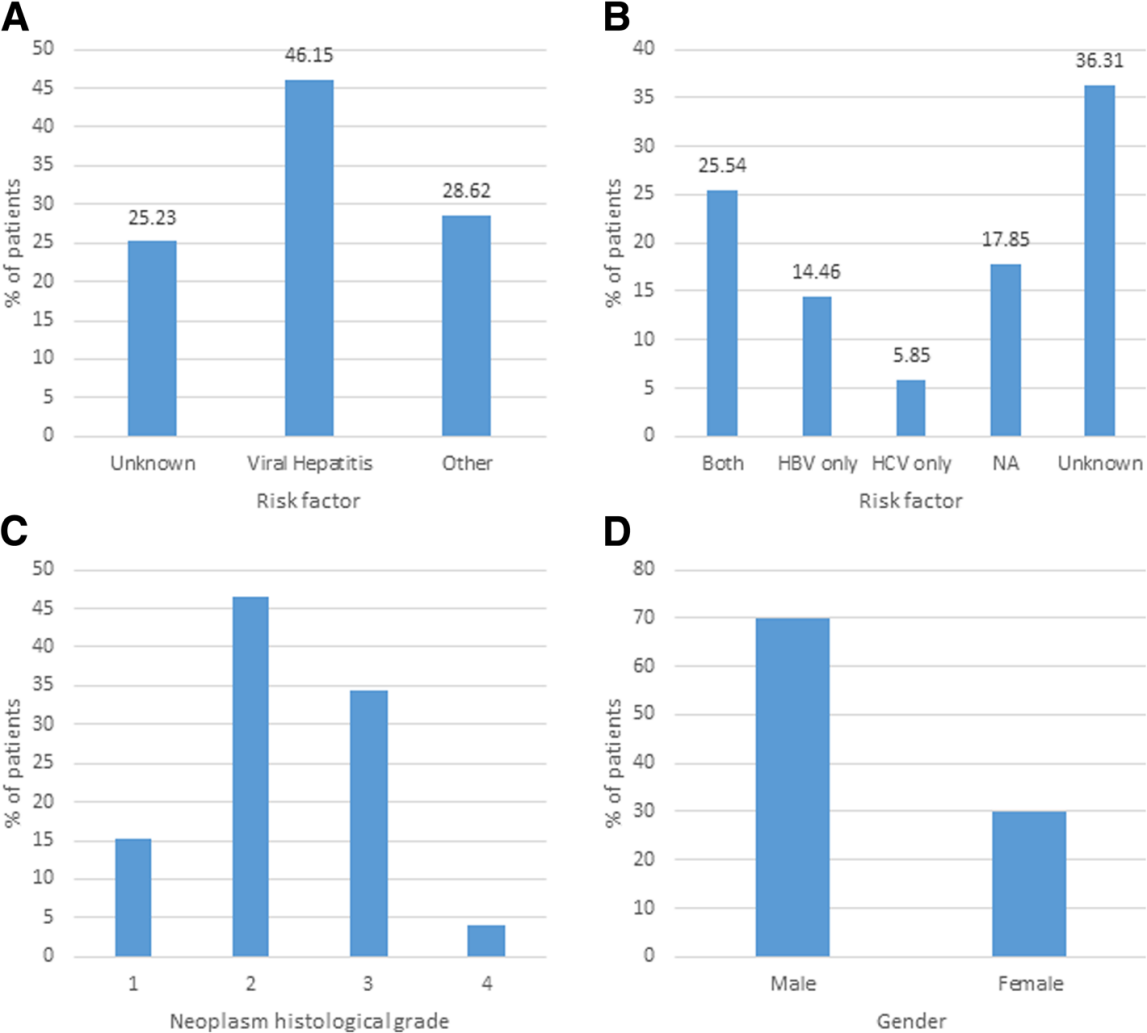
Demographic characteristics of the cancer samples. **a** Risk factors; **b** Detail of Viral Hepatitis risk factors; **c** Neoplasm histological grade; **d** Gender

The dataset was obtained from The Cancer Genome Atlas (TCGA) Research Network [10] and tested under the TCGA approved project #9811. TCGA generated the Illumina exome data from 11,079 patients and 34 different cancer types, including 376 patients with Hepatocellular Carcinoma (HCC). For detailed information on the clinical definition of HCC please refer to The Cancer Genome Atlas (TCGA) Research Network [10]. Figure 1 shows demographic characteristics of the HCC samples. For non-cancer controls (NC), data were obtained from the 1000 Genomes project [11]. This project holds UDS data from 2504 individuals of 26 human populations. From these, 293 samples were selected that satisfied the following criteria: (i) unrelated to each other; (ii) collected from same geographic regions as the HCC samples; (iii) same technology (Illumina) and same exome library preparation as HCC (Nimblegen); (iv) mtDNA genome coverage > 95%, and (v) overall match to HCC samples by gender and mtDNA lineages.

### Pre-processing

**Fig. 2:**
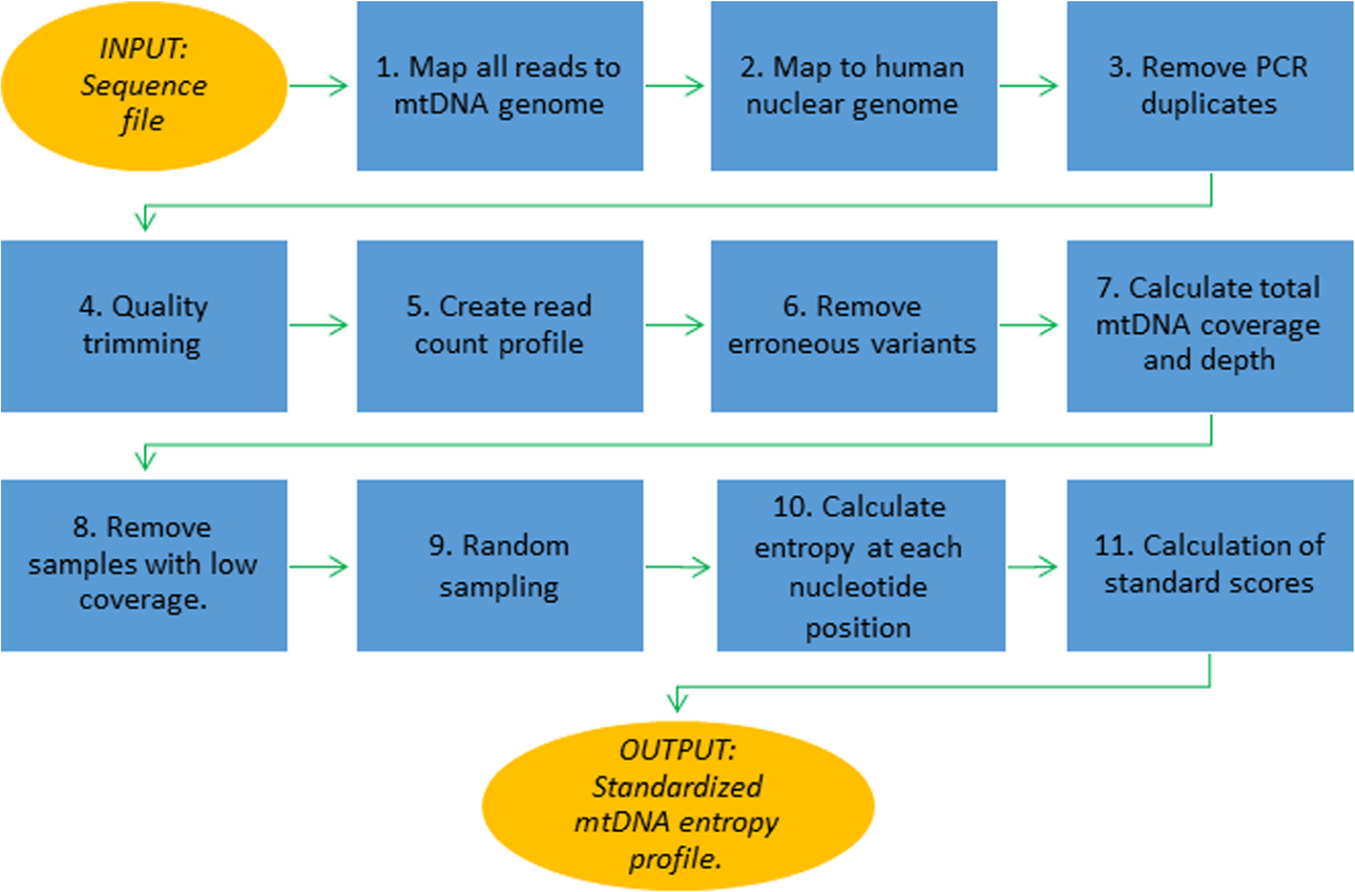
Outline of the pre-processing of sequence files

Figure 2 shows the bioinformatics pipeline implemented here for pre-processing of sequence files. The input is an exome sequence file in the fastq format. The output is a standardized mtDNA entropy profile (SMEP). The pipeline is implemented in Python and optimized to run on a Linux cluster, taking in average of 30 min per set of 16 samples. The pipeline includes the following steps:

1. Reads were mapped to the mtDNA reference [12] using recommendations and parameters implemented in MTOOLBOX [13, 14]. Reads mapped to mtDNA were retained for further analyses.
2. Reads were further mapped to nuclear human genome to remove NUMTs (nuclear mitochondrial DNA segments) following the recommendations and parameters described in [13, 14].
3. PCR duplicates were removed using Picard MarkDuplicates (http://broadinstitute.github.io/picard/)
4. Quality trimming was performed using FAQCS [15].
5. A read count profile was created using BAM-read Count (https://github.com/genome/bam-readcount).
6. Low frequency variants were separated from Illumina sequence errors following the procedure described in [16]. A variant is removed if the probability that it is an error was > 0.00001.
7. The total mtDNA coverage and the depth at each position were caHCCulated.
8. Samples with a total coverage of < 95% of mtDNA are removed.
9. To reduce differences in genetic heterogeneity among files that were solely due to sampling depth, 100 random samples of 50 reads were taken at each mtDNA position. The target number of reads was chosen as this was the average depth found in the HCC dataset (*n* = 49.6).
10. Genetic heterogeneity at each nucleotide site for each of the 100 random samples was caHCCulated as an average of Shannon entropy [17] over all random samples. The Shannon entropy *H* of a nucleotide site *j* with *n* different variants is given by:

$$ {H}_j=-{\sum}_{i=1}^n{x}_i{\mathit{\log}}_b{x}_i $$

Where x_i_ is the fraction of reads covering that position that show variant _i_ and *b* is the base of the logarithm (in this case, b = 2).

11. Finally, to make the profiles more comparable and, thus, to increase the generalization power of the test, we transformed each heterogeneity profile into a set of Z-scores, the signed number of standard deviations by which each observation is above or below the mean of the sample. We found that this standardization greatly improved the accuracy of the classifier.

### Comparison of mtDNA from liver and blood of HCC patients

The samples available from HCC patients included tumor (*n* = 358), normal liver (*n* = 85) and blood (*n* = 293). We performed a detailed comparison of the mtDNA variants showed in the liver and blood of the same HCC patients, in terms of average number of reads, average depth of mtDNA sequencing, total mtDNA entropy, percentage of the mtDNA genome covered, percentage of all reads that map to mtDNA, and number of polymorphic positions. The purpose of this comparison is to show the extent of mtDNA changes observed in tumors and (ii) the degree of homogeneity of these mutations among HCC patients.

### Machine learning

After obtaining SMEP for each sample, we studied the association between SMEP and HCC/NC using the following steps:

1. We measured how the association level of each of the 16,569 nucleotide sites with the HCC/NC grouping my means of Iterative Relief [18, 19]. Machine learning was repeated with different percentages of the 16,569 mtDNA sites (top 1%, 5, 10 …, 95 and 100%), with the top 1% showing the best results (1% =166 sites).
2. Supervised machine learning was performed using the Random Forest (RF) technique [20] as implemented in Sci-kit [21]. Although other methods, such as Nearest Neighbors, Nearest centroid, Support Vector Machine, Logistic regression, Gaussian Naïve Bayes, Decision trees and a Perceptron, were also tested, RF identified best genetic associations to the HCC and NC groups.
3. A grid search of the best combination of parameters was performed. The performance of each combination of parameters was measured using the 10-fold cross-validation (10xCV). The final parameters of the classifier were the following: Number of trees: 101; Maximum tree depth: 4; Minimum number of instances to perform a split: 19. Splitting criterion: entropy; minimum number of instances in a leaf: 1; class weight: balanced.
4. Classifier with the highest 10xCV accuracy was used to test a validation dataset that was not used for the parameter optimization.

In addition, we also tested the most heterogeneous mitochondrial genomic regions, HVS1 (positions 15,977–16,391 bp), which has been extensively used in many genetic studies. Reads covering this region were extracted from the exome data and used to generate the RF classifier using the same procedure described above.

## Results

### mtDNA from liver and blood of HCC patients

**Fig. 3:**
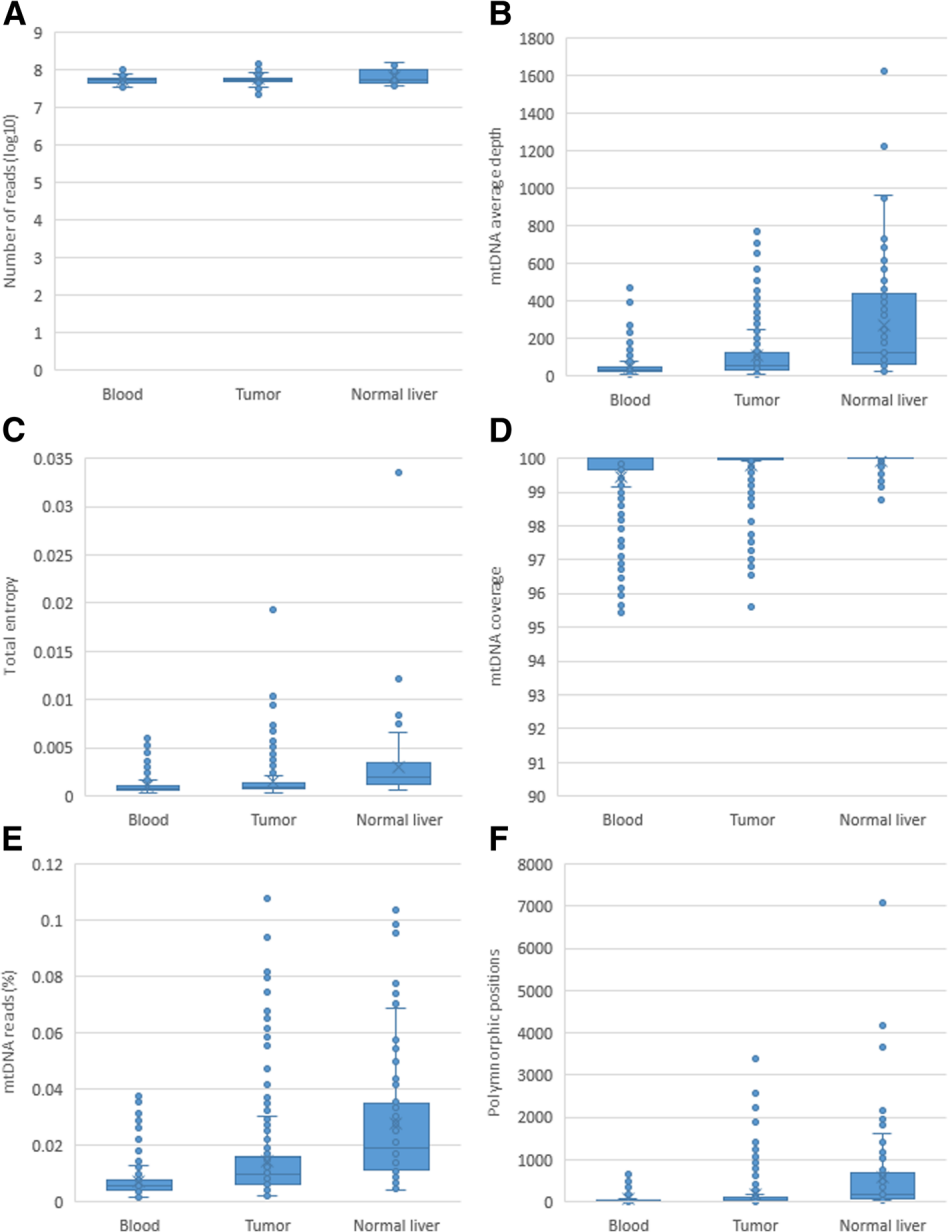
Comparison between tissues of cancer patients. **a** Number of reads, all pairwise comparisons have a *p* value; **b** mtDNA average depth; **c** mtDNA total entropy; **d** Percentage of the mtDNA genome covered; **e** Percentage of all reads that map to the mtDNA genome; **f** Number of polymorphic sites

Figure 3 shows comparison among the samples’ average number of reads, average depth of mtDNA sequencing, total mtDNA entropy, percentage of the mtDNA genome covered, percentage of all reads that map to mtDNA, and number of polymorphic positions. Pairwise comparison among 3 tissues in each HCC patient showed that, with the exception of the number of reads, the above parameters are significantly higher in normal liver (paired t-test; *p* < 0.05), while the lowest values were detected in blood (Table 1), indicating a lower representation of mtDNA in blood as compared to liver and reduction in mtDNA in tumor as compared to normal liver.

**Table 1:** Comparison between tissues of HCC patients. Ratio of the averages and *p* value of the paired samples t-test

	Blood vs Normal liver	*p* value	Blood vs Tumor	*p* value	Normal liver vs Tumor	*p* value
Number of patients	25	N/A	277	N/A	81	N/A
Number of reads (log10)	1.02	8.78E-01	0.99	7.82E-01	1.02	7.82E-01
mtDNA average depth	0.14	9.96E-05	0.49	8.30E-12	1.86	6.68E-04
mtDNA total entropy	0.49	1.40E-03	0.75	2.22E-03	1.26	2.44E-01
Percentage of the mtDNA genome covered	0.98	1.06E-06	1.00	1.95E-06	1.00	4.87E-04
Percentage of all reads that map to the mtDNA genome	0.19	1.45E-06	0.50	2.02E-12	1.72	3.33E-04
Number of polymorphic sites.	0.18	3.17E-04	0.50	8.29E-04	2.09	2.52E-03
Number of different sites (*p* < 0.05)	468	N/A	319	N/A	492	N/A

Consensus sequences of mtDNA were generated for each tissue in each of the samples. On average, the consensus sequences of mtDNA found in tumors and blood of same patient differ at 0.92 sites, being identical in 42.23% of the patients. Consensus sequences from tumor and normal tissue differ at 1.17 sites, being identical in only 37.04% of the individuals. Consensus sequences from blood and normal liver tissue of the same patient were much more similar, with an average difference at 0.16 sites and consensus sequences being identical in 84% of patients.

Pairwise comparison of UDS data from the three tissue samples identified 492 sites, entropy of which was significantly different between tumor and normal liver (paired t-test; *p* < 0.05). However, only 38 of the sites differed between tumor and blood (paired t-test; *p* < 0.05), while blood and tumor mtDNA differ at 319 sites (*p* < 0.05). Despite significant similarity of consensus sequences, entropy of 468 sites differed in mtDNA from blood and normal liver (*p* < 0.05), indicating differences in intra-host mtDNA heterogeneity between these two tissues.

**Fig. 4:**
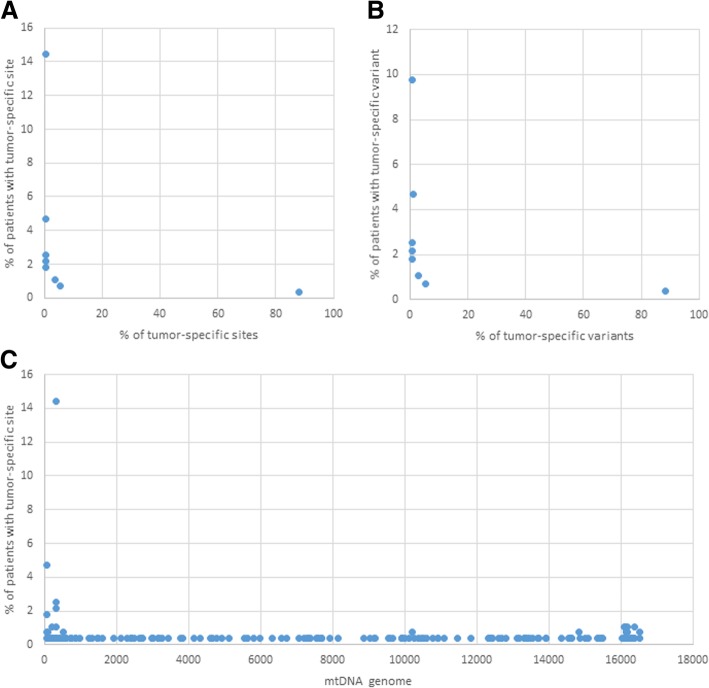
Tumor-specific sites and variants in different HCC patients. **a** Percentage of tumor-specific sites that are present in several HCC patients; **b** Percentage of tumor-specific variants that are present in several HCC patients; **c** Distribution of tumor-specific sites along the genome

The consensus sequences from tumor and blood differed at 169 sites (“tumor-specific” sites) scattered across the entire genome (Fig. 4c). Mutations at these sites (“tumor-specific” mutations) were, however, present at low frequency in the blood of 7.03% of patients and in 18.95% of patients with a normal liver. Most of the tumor-specific mutations (88.16%) were found only once in other HCC patients. Only one tumor-specific mutation at site 310 was present in 14.44% of the HCC patients (Fig. 4a). Both observations indicated a low association of these mutations with HCC.

### mtDNA in HCC and NC patients’ blood

Considering that mtDNA was tested in blood from all cases studied here, analyses on genetic differences in mtDNA between HCC and NC were focused on data from blood. The number of available samples, gender and mtDNA lineages between the HCC and NC groups were equalized to ensure statistical significance of observations on differences between these two groups. The two groups showed small but statistically significant differences in average entropy of mtDNA, percentage of exome reads mapped to mtDNA, percentage of all reads mapped to mtDNA, and number of polymorphic sites (Table 2).

**Table 2:** Comparison between HCC and NC samples. Ratio of the averages and *p* value of the paired samples t-test

	HCC	NC	Ratio	*p* value
Number of reads (log10)	7.7429	7.4892	0.9672	2.62E-15
mtDNA average depth	49.6176	120.0060	2.4186	1.73E-12
mtDNA total entropy	0.0011	0.0014	1.2798	1.96E-05
Percentage of the mtDNA genome covered	99.4562	99.6885	1.0023	0.004217
Percentage of all reads that map to the mtDNA genome	0.0073	0.0388	5.3349	2.78E-30
Number of polymorphic sites.	68.4334	129.8362	1.8973	5.86E-06

**Fig. 5:**
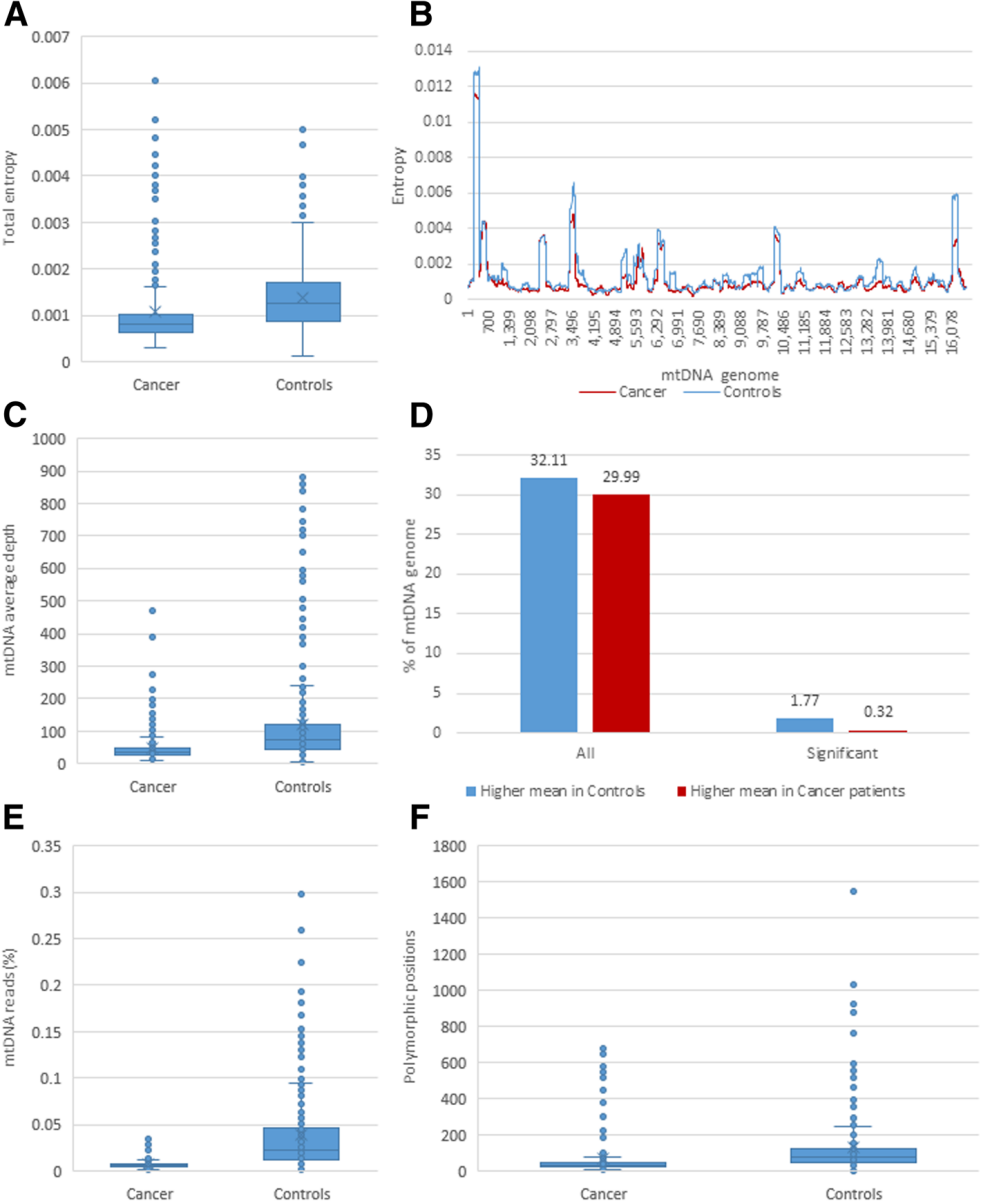
Differences between HCC and NC samples. **a** Average entropy; **b** Average entropy over the mtDNA genome. Sliding moving window = 201 bp, step = 1; **c** Percentage of all exome reads that map to the mtDNA genome; **d** Percentage of mtDNA sites with high average entropy; **e** Percentage of all reads that map to the mtDNA genome; **f** Number of polymorphic sites

When compared with NC, HCC have 1.24-times lower average total entropy (*p* = 2.84E-47) and 3.6-times lower percentage of all reads mapped to mtDNA (*p* = 8.23E-19) (Table 2 and Fig. 5). Among all mtDNA polymorphic sites, 2.09% showed a significantly different mean entropy between HCC and NC. These selected sites were evenly distributed across the entire mtDNA. Only 0.32% of the sites had a higher mean entropy (*p* < 0.05) but 1.77% had a lower mean entropy in HCC (*p* < 0.05). Thus, certain polymorphic sites scattered along mtDNA differed in the degree of diversity between HCC and NC patients, indicating their potential application as markers of HCC.

### Genetic association with HCC

**Fig. 6:**
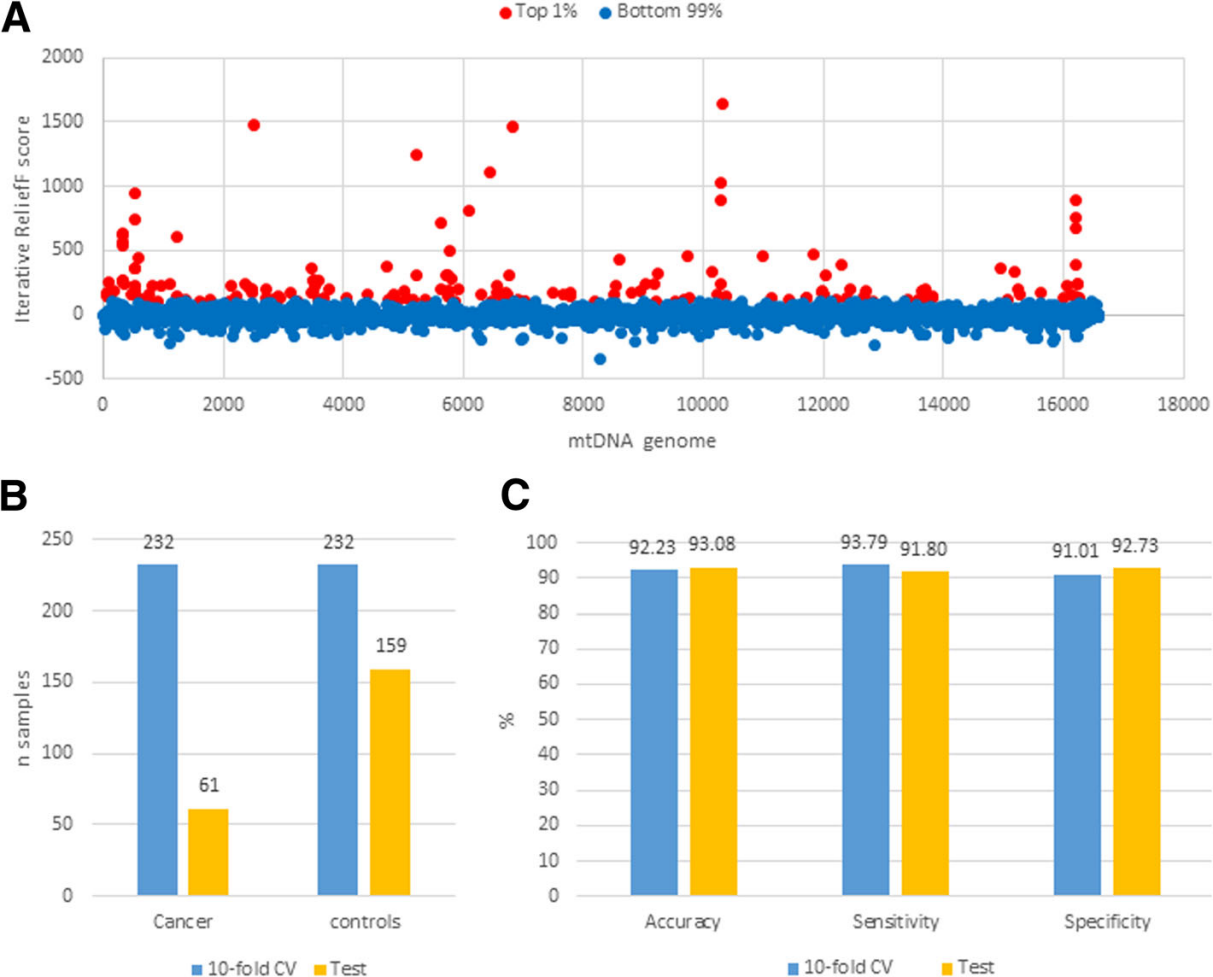
Machine learning results. **a** Importance of each nucleotide position entropy in separating cancer and control samples. Only the sites within the top 1% scores (in red) were used for machine learning; **b** Distribution of samples; **c** Accuracy of the Random Forest classifier

The top 1% of the mtDNA 16,569 nucleotide sites (*n* = 166) with the highest Iterative Relief scores were used for the classifier optimization (Fig. 6a). These sites were not clustered in any gene but spread over mtDNA. The samples were separated into two groups, the first was used for the classifier optimization in 10-fold Cross-Validation (10xCV), and the second, which was not used for the optimization, was used for the final classifier testing. Figure 6b shows the number of samples in each set. Using the first set, the RF-based classifier showed accuracy of up to 99.78% and an average accuracy in 10xCV of 92.23%. Finally, the RF classifier yielded an accuracy of 93.08% on the test dataset (Fig. 6c). All these data indicate that the mtDNA heterogeneity is strongly associated with HCC and NC.

Among the top 1% HCC-specific sites (*n* = 166) selected by ReliefF, only 11 (6.6%) are shared with the “tumor-specific” sites (*n* = 169) selected using consensus sequences. Thus, although both are scattered across the entire mtDNA, individual sites from both groups are very different.

### HVS1 association with HCC

Although polymorphic sites of significance found here are distributed along the entire mtDNA, we tested the performance of only the most heterogeneous mitochondrial genomic region, HVS1. The average 10xCV accuracy was 83.22%, indicating that, although at the reduced rate, the distribution of sites’ entropy in this region alone is strongly associated with HCC. However, it should be noted that increase in the coverage depth might help to identify more polymorphic sites in this region, thus potentially improving accuracy of classification. Application of UDS to a small genomic region could offer a greater control over sequencing depth, which is important for accurate assessment of genetic heterogeneity, especially in mtDNA extracted from blood where its concentration is low.

### Assessment of the neoplasm histologic grade

In the TCGA HCC dataset, 292 samples had available information regarding the neoplasm histologic grade: 41 samples were classified as stage 1 cancer, 134 samples were stage 2, 104 samples were stage 3 and 13 samples were stage 4 (Fig. 2c). In 10xCV, the RF Regression yielded an average absolute error of 0.61, which is only 2X better than the average absolute error of a random assignment (1.249), showing only a moderate association of SMEPs with the grades. Implementation of binary classification schemes instead of regression (e.g. Stage 1 vs all others) didn’t improve classification accuracy.

## Discussion

Analyses conducted in this study indicate that heterogeneity profiles of the intra-host mtDNA variants from blood are strongly associated with HCC. Although cancer detection is usually focused on genetic analysis of nuclear DNA [2], mtDNA has been shown to be functionally associated with several cancer types [22]. Owing to its clonal nature, high copy number and high mutation rate [23], mtDNA has many practical advantages over nuclear DNA in application to cancer detection. Mitochondria supply energy for all metabolic processes and control apoptosis, and as such are essential for multiplication of cancer cells. The mitochondrial oxidative phosphorylation system has a major effect on tumor progression [22, 24]. In addition, enhanced progression to malignancy was observed in cells with compromised mitochondrial integrity [22, 24]. mtDNA mutations are significantly associated with the development of various types of cancer (for a review see [22]).

Clonal expansion of mutant mtDNA species was reported in 27–80% (average 54%) of malignant tumor samples (for a review see [25]). In concert with this observation, we found that consensus sequences of mtDNA differ between tumor and blood from ~ 58% of patients. Both particle-associated and free mtDNA are present in blood [26], potentially providing a convenient and minimally invasive way for the detection of cancer-related mitochondrial mutants [7]. As many cancer types, HCC is associated with clonally expanding mtDNA mutations [27–33]. The clonal expansion should affect genetic composition of mtDNA variants in blood. However, such an effect is not straightforward because mtDNA in blood has a very complex origin [26]. Moreover, requirements for efficient energy supply to rapidly replicating malignant cells constrains genetic composition of mitochondria in tumors [34].

The clonal expansion and genetic constraints coupled with a small size (16,569 bp) make mtDNA especially suitable for the accurate assessment of association of intra-host genetic heterogeneity, rather than specific mutations, with cancer. Application of heterogeneity profiles implemented here to the HCC detection overcomes the often-idiosyncratic presentation of specific mutations in cancer. Indeed, most tumor-specific variants (99.4%) found in this study were present in less than 5% of HCC patient, thus impeding their use as general cancer markers. Complex and variable genetic nature of cancer is well established. It hinders the identification of specific mutations suitable for cancer diagnostics [3, 35, 36]. However, measures of intra-host genetic diversity in place of specific states of nucleotide sites mitigate the contribution of host-specific genetics to the detection of associations with cancer.

Tumor-specific mutations were present at low frequency in the blood of only 7.03% of patients. This finding indicates that the direct contribution of the tumor to the genetic composition of mtDNA in blood is limited, thus potentially confounding the detection of tumor-specific genetic variants in blood for cancer diagnostics. This concern becomes especially relevant when one considers a significant drop in mtDNA load in blood observed in this study and also reported elsewhere [37]. Nevertheless, the RF-classifiers generated here separated HCC and NC patients with accuracy exceeding 93%, indicating the existence of a strong HCC-specific genetic signal in intra-host mtDNA populations.

Genetic factors used in the RF-classifiers are fundamentally different from tumor-specific mutations identified from consensus mtDNA sequences. Only 11 tumor-specific sites were among the top 166 sites selected by entropy as relevant to the HCC/NC classification, despite the fact that both sets of sites scattered along the entire mtDNA. Site entropy or its Z-score do not have information on a specific nucleotide state of a site, rather both measure nucleotide diversity at each site, thus reducing strong effects of specific mutations on associations captured by our models. There are many genetically diverse lineages of mtDNA. Although the HCC and NC datasets were matched by geographic location and mtDNA lineages, genetic differences among different genetic types of mtDNA may impede the identification of cancer-specific mutations, especially in a limited dataset. Entropy, however, represents a more general genetic information that can adequately trim genetic differences among mtDNA lineages, focusing nucleotide heterogeneity analyses on the identification of other than lineage-specific traits. Models generated using Z-scores performed as well as the entropy-based models. However, contribution of standardization achieved by application of Z-score to accuracy of models may become more apparent on more heterogeneous datasets.

Here, we applied machine-learning algorithms to extract genetic information from mtDNA for discriminating between HCC and NC. Application of the algorithms is routine in industrial and technological applications and only recently became successfully explored in clinical field [38, 39]. Machine learning presents a new opportunity to cancer diagnostics by shortcutting research from learning molecular mechanisms before developing applications to direct identification of reliable markers, thus accelerating development of accurate cancer detection.

We showed that tumor-specific mutant mtDNA species may be present at a very low concentration in blood. The detection of such minority variants can be achieved by UDS. Indeed, UDS has been applied to the efficient detection of tumor DNA [8] and to the detection of minority cancer-specific DNA variants [9]. However, a significant depletion of mtDNA has been reported for several cancer types such as bladder, breast, kidney, and liver cancer [37] making the detection of minority tumor-specific variants especially challenging. In agreement, we observed a ~ 2-fold decline in the number of reads mapped to mtDNA from tumor as compared to normal liver tissue, which further emphasizes potential difficulties in identification of specific mutant variants in tested blood. These observations indicate that consistent detection of minority variants is strongly contingent to a very high depth of sequencing. However, in difference to the detection of specific mutations, accurate estimation of site heterogeneity can be done at a moderate sequencing depth, thus providing a more reliable source of cancer-specific markers.

Uniform and adequate read coverage of the entire mtDNA can be challenging for the shotgun-based UDS. Sequencing of a single amplicon offers a greater control over the read coverage. However, it limits the mtDNA presentation to a single genomic region. Taking these observations in consideration, we hypothesized that such highly heterogeneous region of mtDNA as HVS1, may have sufficient genetic information to identify association with HCC. Indeed, the model constructed using HVS1 alone identified HCC versus NC with 83.22% 10xCV accuracy, indicating its applicability to the detection of HCC.

Finally, the observations presented here indicate significant differentiation of mtDNA heterogeneity between HCC and NC patients. Although showing separation between these 2 groups of patients, the data, however, do not allow to ascertain the strict HCC specificity of the classifiers. Detection of specific types of cancer versus general malignancy warrants additional investigation.

## Conclusions

Sites contributing most to the association with HCC are scattered along the mtDNA genome, affecting all mitochondrial genes. The findings suggest that application of heterogeneity profiles of intra-host mtDNA variants from blood overcomes the complex association of specific mutations with cancer for the development of accurate, rapid, inexpensive and minimally invasive diagnostic detection of cancer.

The findings in this study suggest that genetic diversity of intra-host mtDNA in blood may serve as a generalizable marker for the accurate, rapid, inexpensive and minimally invasive diagnostic detection of cancer.
